# Supplementary figures and images for: DNA Barcode, chemical analysis, and antioxidant activity of Psidium guineense from Ecuador
Source: PLoS One. 2025 Mar 19;20(3):e0319524. doi: 10.1371/journal.pone.0319524 (PMC11922285; doi:10.1371/journal.pone.0319524)

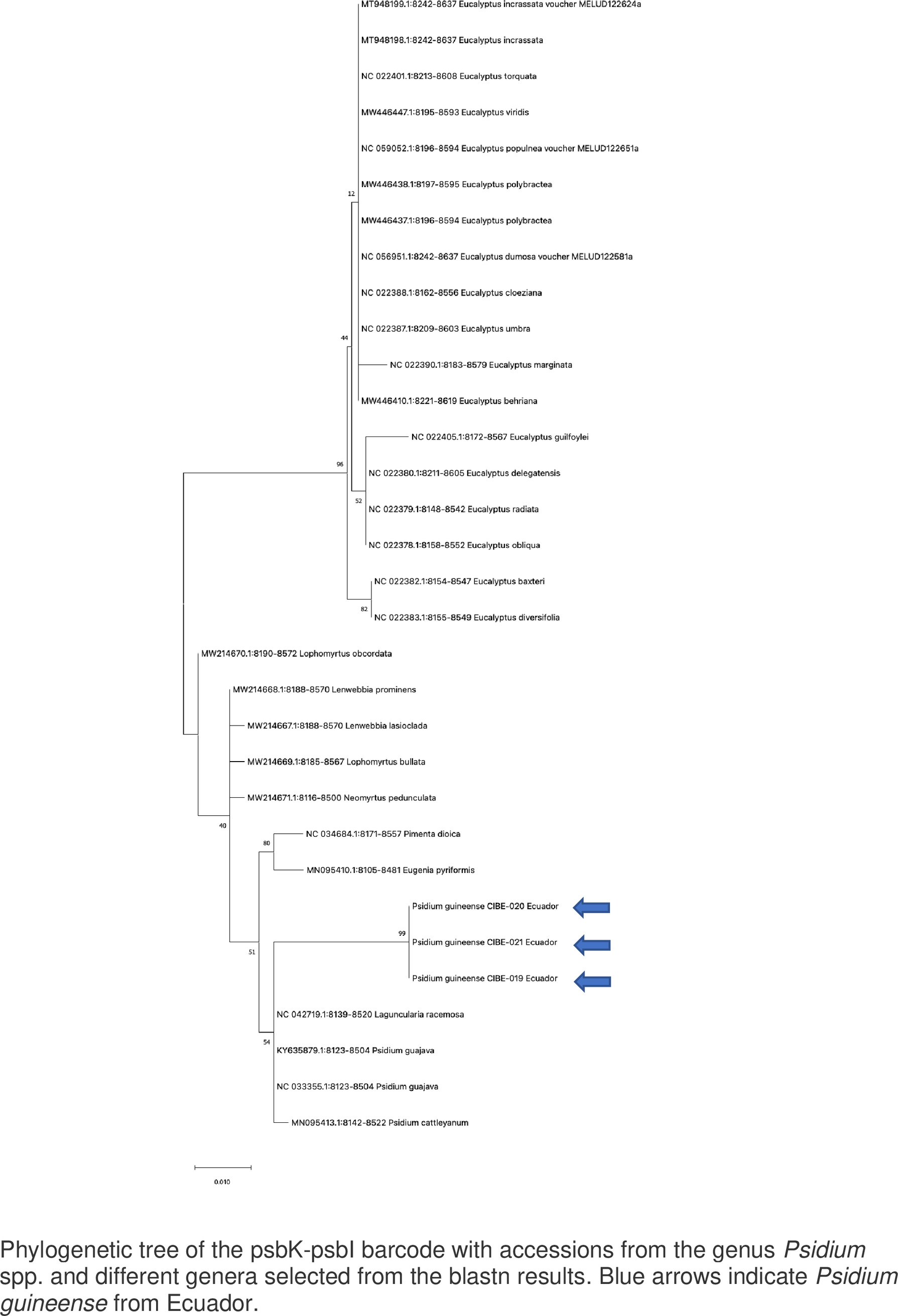

Supplement: S1 Fig — (TIF) [file pone.0319524.s002.tif]
