# Supplementary material for: Correction: DNA Barcode, chemical analysis, and antioxidant activity of Psidium guineense from Ecuador
Source: PLoS One. 2025 Jun 5;20(6):e0326074. doi: 10.1371/journal.pone.0326074 (PMC12140653; doi:10.1371/journal.pone.0326074)
Supplement: S2 File — (PDF) [file pone.0326074.s002.pdf]

PLOS ONE

RESEARCH ARTICLE

DNA Barcode, chemical analysis, and antioxidant activity of *Psidium guineense* from Ecuador

Joel Eduardo Vielma-Puente[1*☯], **Efrén Santos-Ordóñez**[2,3☯], **Xavier Cornejo**[4‡], **Iván Chóez-Guaranda**[2‡], **Ricardo Pacheco-Coello**[2‡], **Liliana Villao-Uzho**[2‡], **Christian Moreno-Alvarado**[1‡], **Natalia Mendoza-Samaniego**[1‡], **Yuraima Fonseca**[5‡]

1 Faculty of Natural Science and Mathematics, ESPOL Polytechnic University, Escuela Superior Politécnica del Litoral, ESPOL, Guayaquil, Guayas, Ecuador, **2** Biotechnological Research Center of Ecuador, ESPOL Polytechnic University, Escuela Superior Politécnica del Litoral, ESPOL, Guayaquil, Ecuador, **3** Faculty of Life Sciences, ESPOL Polytechnic University, Escuela Superior Politécnica del Litoral, ESPOL, Perimetral, Guayaquil, Ecuador, **4** Departamento de Botánica, Facultad de Ciencias Naturales, Universidad de Guayaquil, Guayaquil, Guayas, Ecuador, **5** Departamento de Química, Facultad de Ciencias, Universidad de Los Andes, Mérida, Mérida, Venezuela

☯ These authors contributed equally to this work.
‡ XC, ICG, RPC, LVU, CMA and NMS, YF also contributed equally to this work.
* jvielma@espol.edu.ec

Check for updates

OPEN ACCESS

Citation: Vielma-Puente JE, Santos-Ordóñez E, Cornejo X, Chóez-Guaranda I, Pacheco-Coello R, Villao-Uzho L, et al. (2025) DNA Barcode, chemical analysis, and antioxidant activity of *Psidium guineense* from Ecuador. PLoS One 20(3): e0319524. https://doi.org/10.1371/journal.pone.0319524

Editor: Hope Onohuean, Kampala International University - Western Campus, UGANDA

Received: August 5, 2024

Accepted: February 3, 2025

Published: March 19, 2025

Copyright: © 2025 Vielma-Puente et al. This is an open access article distributed under the terms of the Creative Commons Attribution License, which permits unrestricted use, distribution, and reproduction in any medium, provided the original author and source are credited.

Data availability statement: All relevant data are within the manuscript and its Supporting Information files.

Funding: Funding was provided by the Research Dean of ESPOL, Project: FCNM-17-2018. Received by the Project Director J. Vielma.

Abstract

This study investigates the phytochemical, genetic, and antioxidant properties of *Psidium guineense*, a species native to the tropical dry forests of Ecuador. Leaves were collected, preserved in recognized herbaria, and subjected to Soxhlet extraction using polar and non-polar solvents. Phytochemical screening revealed the presence of secondary metabolites, while GC-MS analysis detected chemical compounds in the extracts. Antioxidant assays demonstrated high phenolic (54.34 ± 0.49 mg GAE/g) and flavonoid (6.43 ± 0.38 mg QE/g) content, with significant antioxidant activity in DPPH (0.57 ± 0.04 mg TE/g), FRAP (105.52 ± 6.85), and ABTS (1.25 ± 0.01 mg TE/g) assays. DNA barcoding of nine loci, (seven from the chloroplast genome and two nuclear genome) using a CTAB extraction protocol and PCR, provides the first genetic characterization of this species, contributing to genetic diversity assessments and phylogenetic studies. These findings underscore the importance of *P. guineense* as a source of potent bioactive compounds with significant antioxidant potential, highlighting its applicability in nutritional and pharmaceutical industries. Additionally, the genetic insights gained support efforts to expand DNA barcoding databases for tropical biodiversity conservation.

Introduction

Ecuador is known as a megadiverse country [1] and has an important number of ecosystems and endemic vascular plant species [2,3]. One of the most important ecosystems is the tropical dry forest, located in the center and south of the western region of Los Andes, in the provinces of Guayas, Esmeraldas, Manabí, Loja, Imbabura, and El Oro [4].

Competing interests: The authors have declared that no competing interests exist.

Species of the genus *Psidium* belonging to the *Myrtaceae* family, are found from Southern Mexico to Northern Argentina and Brazil [5] and are widely distributed in the Ecuadorian tropical dry forest, some species are considered endemic and could be of great interest in the pharmaceutical industry. Many Ecuadorian endemic plants are used traditionally by natives like medicinal plants to treat diseases [6].

Psidium guineense, commonly known as Brazilian guava or guayabilla, is a small tree or shrub native to tropical dry forests and mesophilic mountain forests [7]. The leaves are evergreen, oblong to ovate, or obovate, measuring between 3.5 to 14 cm in length and 2.5 to 8 cm in width. The upper surface of the leaves is somewhat hairy, while the underside is covered with pale or rusty hair and dotted with glands, giving them a greyish-green color. Flowers are white, typically borne singly or in clusters of three in the leaf axils, with numerous prominent stamens (150 to 200) that give them a bushy appearance [8]. The fruit is small, round, or pear-shaped, measuring between 1.0 to 2.5 cm in diameter, with a yellow skin and pale-yellowish flesh surrounding a white central pulp. The flavor is slightly acidic and resinous, reminiscent of strawberries or pineapple jam, and each fruit contains numerous small, hard seeds dispersed by birds and mammals [8].

Psidium guineense grows best in temperatures ranging from 23°C to 28°C (73°F to 82°F) and is sensitive to frost, with young trees affected at temperatures below -3°C (27°F) [9]. The species is adapted to a range of altitudes, from coastal areas to higher terrains, and can grow in various soil types, making it capable of thriving in diverse environmental conditions [10].

Psidium species have been studied and shown pharmacological potential, reported in the treatment of diseases of diarrhea [11,12], dysentery [12], digestive system diseases [13], stomach pain [12], antimalarial [14], antiplasmodial [15], anti-inflammatory [16], antiparasitic [17], antimicrobial [16], larvicide [16] and others. Additionally, a high antioxidant potential has been documented in different species of the genus *Psidium* such as, *Psidium guajava* [16], *Psidium friedrichsthalianum* [18], *Psidium cattleianum* [19,20], *Psidium guineense Swartz* [21], *Psidium acutangulum* [22], *Psidium laruotteanum* [23], *Psidium salutare* [24], *Psidium sobralianum* [24], *Psidium bahianum* [25] is known.

Oxidative stress is a major factor in the development of many diseases such as diabetes [26,27], cancer [27], cardiovascular [28], neurological [29], and respiratory diseases [30].

Antioxidants have been proven to have an essential role in diseases by clearing reactive species in the body [31]. Polyphenols are known for their antioxidant properties [32], have gained significant attention in recent years as natural compounds that can mitigate oxidative stress. The genus *Psidium* is particularly notable for its chemical composition, which includes a wide variety of polyphenols [32,24], flavonoids [33], tannins [34], alkaloids [33], triterpenes [33], carotenoids [33], carbohydrates [20], fatty acids [20], and other bioactive compounds [35]. These components contribute to the genus pharmacological potential, including antioxidant activity, which is critical in addressing oxidative stress-related conditions.

Previous studies have identified that *P. guineense* leaf extracts possess bioactive compounds such as phenolics, and flavonoids [36,37]. Their antioxidant, anti-inflammatory, antiproliferative, and antimycobacterial properties have also been evaluated [36–39]. The compounds may vary in their levels of bioactivity due to the environmental conditions to which each plant was exposed [40].

On the other hand, DNA barcodes could offer several benefits. This methodology is more accurate than morphological characteristics for identifying plant species, and more importantly, it could serve as a complement analysis for taxonomic identification. This is mainly because many plant species appear similar but could differ genetically. DNA barcodes allow scientists to overcome this problem and accurately and cost-effectively identify plant species. DNA barcoding can aid in conserving rare and endangered species and support research on

plant evolution, ecology, and conservation, especially given the threats to biodiversity from human activities, pollution, deforestation, and resource depletion [41].

Thisstudy aims aphylogenetic identification of*Psidium guineense*, analyze its chemical composition, and evaluate its antioxidant activity to assess its pharmaceutical potential. The study involve a comprehensive analysis of the chemical composition, phenolic and flavonoid content, and antioxidant activity of *Psidium guineense,* a species native to the tropical dry forests of Ecuador. Furthermore, for the first time, DNA barcodes were determined for nine *loci* (seven from the chloroplast genome and two for the nuclear genome). This achievement represents a significant fact for recognizing the phylogeny of the species, tracing its evolutionary origin, and predicting potential genetic variations.

Methods

Reagents

Methanol (99.9%), ethyl acetate (> 99.5%), dichloromethane (99.7%), and hexanes (95%) were obtained from Fisher Chemical. Hydrochloric acid (10 N) and ferric chloride hexahydrite (97%) were obtained from Fisher Scientific. 2,2,2-trifluoro-N-(trimethylsilyl) acetimidate (BSTFA) (98%), and Trolox (97%) were obtained from Thermo Scientific™. 2,2-Azino-bis(3-ethylbenzothiazoline-6-sulfonic acid) diammonium salt (ABTS) (99.4%) was obtained from Thermo Fisher Scientific. Folin & Ciocalteu´s phenol reagent was obtained from OttoKemi (2 N). Sodium carbonate anhydrous (99.5%), aluminum chloride (99%), 2,2-diphenyl-1pycrilhydrazyl (DPPH) (95%), potassium acetate (99%), and quercetin hydrate (97%) were obtained from Merck. Gallic acid (98%) was obtained from Titan Biotech. 2,4,6-tri(2-pyridyl)-1,3,5-triazine (TPTZ) (98.5%) was obtained from J&K Scientific. All reagents were used without prior purification.

Plant material

Leaves from *P. guineense* were collected on July 16, 2022, during the dry season in Colimes, Guayas Province-Ecuador (1°32´S, 80°0´W); the samples were collected in areas not protected by government entities; therefore, collection permits were not required. A part of them was frozen for DNA extraction and another part was cut into 1x1 cm squares, dried in an industrial try-dryer to 60°C, with airflow during 8 h, and used for extraction with different solvents. Voucher specimens are deposited in the Herbario GUAY, Faculty of Natural Sciences, University of Guayaquil, Ecuador, and New York Botanical Garden (NYBG) STEERE HERBARIUM, New York, USA [42].

Morphological analysis

Measurements are from the field, cultivated and GUAY herbarium specimens, morphological observations were performed with a stereoscope Olympus SZ51. The botanical terms used in the description of species follow Jackson B.D. [43].

Solvent extraction by Soxhlet apparatus

Chemical compounds were extracted using polar solvents (methanol, ethyl acetate, and dichloromethane) and a non-polar solvent (Hexane). Experiments were conducted to qualitatively and quantitatively analyze the chemical compounds present in the leaves of the *Psidium* species under study. In the Soxhlet apparatus, 20 g of dry leaves and 200 mL of solvent were used. The extraction was performed for 2 hours at the reflux temperature of different solvents. The extract obtained was filtered and stored in a refrigerator at 3°C [44].

Qualitative phytochemical screening

For the phytochemical screening, 30 g of dried material was mixed with 90 mL of distilled water and macerated for 48 hours. The aqueous extracts were then divided into fractions for subsequent determination of secondary metabolites that might be present in the aqueous extract [45].

GC-MS analysis

P. guineense extracts were analyzed in a gas chromatography-mass spectrometry equipment Agilent Technologies (7890A GC system and 5975C inert XL MSD with triple axis detector) as described in previous studies [46]. A fused-silica capillary column DB-5MS (30 m × 0.25 mm) with phenyl dimethylpolysiloxane was used as the stationary phase (0.25-micron film thickness), and helium as the carrier gas (1.2 mL/min). Hexane, dichloromethane, and ethyl acetate extracts were injected directly. Methanol extracts were dried using a vacuum centrifuge concentrator, mixed with BSTFA, and incubated in a water bath at 80°C for 2 hours. After that, 1 μL of the samples was injected using the splitless mode at 250°C. The oven temperature was started at 70°C for 2 minutes, then it was increased to 285°C at 5°C/min. The MSD transfer line was 300°C, and the ion source temperature was 230°C. An electron ionization of 70 eV was used, and the compounds data was collected with the full scan mode (40-600 amu) in the quadrupole mass analyzer. Finally, compound identification was done by matching the mass spectra information with data available in Wiley 9, and NIST 2011 libraries.

DNA extraction and PCR

Leaves from collected samples from three specimens (biological replicates, coded as CIBE-019, CIBE-020, and CIBE021) were ground using liquid nitrogen in the grinder MM400 (Retsch, Haan, Germany) and stored at −80°C upon DNA extraction.

Approximately, 100 mg of leaf was used for DNA extraction using a CTAB protocol with some modifications [47]. PCR was conducted using the 2× GoTaq master mix (Cat. # M7123; Promega, Madison, WI, USA) using 0.5 μM of each primer (Table 1). The final volume was 30 μL per reaction. PCR conditions consisted of first 95°C for denaturation; followed by 35 cycles of: 95°C for 30 s, 60°C (for rbcL, psbA-trnH, rpoC1), 56°C (for matK, psbK-psbI, atpF-atpH, and ITS2), or 50°C (for rpoB and ITS1) for 30 s, 72°C for 90 s, with a final extension of 72°C for 5 min. Five microliters of PCR reaction were loaded on a 1.5% gel to check for the presence of amplicons. The remaining 25 μL were sequenced commercially after purification of PCR product (Macrogen, Rockville, MD, USA). At least two technical replicates were sequenced, and a consensus was constructed for each biological replicate.

Bioinformatics analysis of sequences

Sequences were trimmed manually using MEGAX after alignment [51]. Processed sequences were blasted (25th September 2023) in the GenBank using the nucleotide database [52]. Sequences from the blast analysis were selected for phylogenetic analysis using MEGAX. For each barcode, the recommended model from the MEGAX was used for phylogenetic analysis after alignment with MUSCLE. The aligned sequences were trimmed at the ends to allow for all sequences to maintain the same range. For the phylogenetic analysis, the Maximum Likelihood method was used for each barcode using a bootstrap test (1000 replicates).

Total phenolic content

The total phenolic content was estimated by the Folin-Ciocalteu method reported by Avramova *et al.* 2017 with modifications [53]. In this procedure, 20 μL of aqueous extract or standard

Table 1. Primers used for PCR amplification of the DNA barcodes *psbA-trnH* spacer, *psbK-psbI* spacer, *rpoB*, *rpoC1*, *atpF-atpH* spacer, *rbc*L, *mat*K, and ITS2.

Locups	Primer pairs	Sequence	Annealing temperature	Reference
psbA-trnH	trnHf_05	CGCGCATGGTGGATTCACAATCC	60°C	[48]
	psbA3_f	GTTATGCATGAACGTAATGCTC		
psbK-psbI	psbK_F	TTAGCCTTTGTTTGGCAAG	56°C	[48]
	psbI_R	AGAGTTTGAGAGTAAGCAT		
rpoB	rpoB_2F	ATGCAACGTCAAGCAGTTCC	50°C	[48]
	rpoB_3R	CCGTATGTGAAAAGAAGTATA		
rpoC1	rpoC1_2F	GGCAAAGAGGGAAGATTTCG	60°C	[48]
	rpoC1_4R	CCATAAGCATATCTTGAGTTGG		
atpF-atpH	atpF_F	ACTCGCACACACTCCCTTTCC	56°C	[49,50]
	atpH_R	GCTTTTATGGAAGCTTTAACAAT		
*rbc*L	rbcLA_F	ATGTCACCACAAACAGAGACTAAAGC	60°C	[51]
	rbcLA_R	GTAAAATCAAGTCCACCRCG		
*mat*K	matK_3F_KIMF	CGTACAGTACTTTTGTGTTTACGAG	56°C	[50,48]
	matK_1R_KIMR	ACCCAGTCCATCTGGAAATCTTGGTTC		
ITS1	ITS 5a F	CCTTATCATTTAGAGGAAGGAG	50°C	[52]
	ITS 4 R	TCCTCCGCTTATTGATATGC		
ITS2	S2F	ATGCGATACTTGGTGTGAAT	56°C	[52]
	S3R	GACGCTTCTCCAGACTACAAT		

https://doi.org/10.1371/journal.pone.0319524.t001

was mixed with 100 µL of 10% (v/v) Folin-Ciocalteu reagent solution, and 80 µL of 7.5% (w/v) sodium carbonate into a 96-well plate. The reaction was incubated for 1 hour in darkness, and the absorbance was measured at 765 nm against a blank in a Biotek Synergy HTX multi-mode microplate reader with a UV-VIS detector (Vermont, USA). The results were expressed as milligrams of gallic acid equivalents (GAE) per gram of dry weight according to the equation $y = 0.0059x - 0.0006$ ($R^2 = 0.9992$) obtained from the standard gallic acid calibration curve (10 – 200 µg/mL).

Total flavonoid content

The total flavonoid content was estimated by the aluminum chloride method reported by Avramova *et al.* 2017 with modifications [54]. For this, 20 µL of aqueous extract or standard was mixed with 10 µL of 10% (w/v) aluminum chloride, 10 µL of 1M potassium acetate, 60 µL of methanol, and 120 µL of distilled water into a 96-well plate. The reaction was incubated for 30 minutes in darkness, and the absorbance was measured at 415 nm against a blank in a Biotek Synergy HTX multi-mode microplate reader with a UV-VIS detector (Vermont, USA). The results were expressed as milligrams of quercetin equivalents (GAE) per gram of dry weight according to the equation $y = 0.0051x - 0.0275$ ($R^2 = 0.9909$) obtained from the quercetin calibration curve (10 – 100 µg/mL).

Antioxidant activity

ABTS radical cation inhibition assay. The ABTS radical cation inhibition activity was measured according to the methodology described by Viteri *et al.* 2022 [54]. An aliquot of 50 µL of the aqueous extract or standard was mixed with 150 µL of a 156 µM ABTS radical cation solution. The reaction was incubated in darkness for 30 minutes and the absorbance was measured at 732 nm against a blank in a Biotek Synergy HTX multi-mode microplate reader with a UV-VIS detector (Vermont, USA). The ABTS radical cation stock solution

was prepared by reacting 7 mM ABTS with 3.6 mM potassium persulfate and incubating it in darkness for 24 hours before use. Then, the stock solution was diluted in water to obtain a final concentration of 156 μM. The results were expressed as milligrams of Trolox equivalents (TE) per gram of dry weight according to the equation $y = 0.5808x - 0.1467$ ($R^2 = 0.9936$) obtained from the Trolox calibration curve (20 – 180 μmol/L).

DPPH radical scavenging assay. The DPPH radical scavenging activity was measured following the procedure described by Viteri *et al.* 2022 with some modifications [54]. Briefly, 50 μL of the aqueous extract or standard was mixed with 150 μL of a 0.1 mM DPPH solution in a 96-well plate. The reaction was incubated in darkness for 30 minutes, and the absorbance was measured at 517 nm against a blank in a Biotek Synergy HTX multi-mode microplate reader with a UV-VIS detector (Vermont, USA). The results were expressed as milligrams of Trolox equivalents (TE) per gram of dry weight according to the equation $y = 0.7853x - 35.622$ ($R^2 = 0.9868$) obtained from the Trolox calibration curve (60 – 180 μmol/L).

Ferric reducing antioxidant power (FRAP) assay. The ferric-reducing antioxidant power (FRAP) was measured according to the methodology described by Hozzein *et al.* 2020 with some modifications [55]. Briefly, 20 μL of the aqueous extract or standard was mixed with 180 μL of FRAP reagent mix in a 96-well plate. The FRAP reagent mix was prepared the day of the reaction using a 10:1:1 (v/v/v) proportion of 300 mM acetate buffer (pH 3.6), 10 mM TPTZ solution dissolved in 40 mM hydrochloric acid, and 20 mM ferric chloride dissolved in water. The reaction was incubated in darkness for 20 minutes, and the absorbance was measured at 600 nm against a blank in a Biotek Synergy HTX multi-mode microplate reader with a UV-VIS detector (Vermont, USA). The results were expressed as milligrams of Trolox equivalents (TE) per gram of dry weight according to the equation $y = 0.0024x + 0.1274$ ($R^2 = 0.9897$) obtained from the Trolox calibration curve (50 – 1000 μmol/L).

Results and discussion

Morphological evaluation

Shrubs to low trees up to 5 meters high, with tomentulose or subtomentose twigs, lower leaf surfaces, and flower buds; young twigs terete to complanate, but not angled. LEAVES simple, opposite, the blade elliptic to oblong, 7–11 x 3–7 cm, the margins entire; apex shortly acuminate to broadly obtuse; base inconspicuously subcordate to broadly cuneate; petiole 4–8 mm long, more or less channeled; venation brochidodromous, the lateral veins 7–11 pairs. FLOWER BUDS conspicuously constricted, ovate to globose in the distal half. CALYX closed in the bud, acuminate at apex, longitudinally tearing in distal half, tomentulose to subtomentose without; petals white, glabrous to pilose without; stamens 150–300. FRUIT obovate to subglobose, 1–3 cm in diameter, epicarp 1–2.5 mm thick.

Qualitative phytochemical screening

The identification of phytochemicals in *P. guineense* leaves is a fundamental starting point for evaluating their nutritional and biological properties. Phytochemical screening detected catechins, saponins, triterpenoids, steroids, flavonoids, alkaloids, reducing sugars, tannins, phenolic compounds, and oils and fats (Table 2). Ethereal, alcoholic, and aqueous extracts had high alkaloid content, these compounds are useful as diet ingredients [56], supplements, and pharmaceuticals [57,58], in medicine and other biological applications.
Table 2 highlights the presence of key bioactive compounds associated with antioxidant activity, particularly in the alcoholic and aqueous extracts. Flavonoids, phenolic compounds, tannins, and catechins—detected predominantly in these extracts—are well-documented for their

Table 2. Qualitative phytochemical screening of *P. guineense* in ethereal, alcoholic, and aqueous extracts.

Compound detected	Inference		
	Ethereal extract	Alcoholic extract	Aqueous extract
Catechins	ND	+	ND
Coumarins and lactones	-	ND	ND
Resins	ND	-	ND
Lactones	ND	-	ND
Oils and fats	+	ND	ND
Saponins	ND	-	+
Quinones	ND	-	ND
Triterpenoids and steroids	+	+	ND
Flavonoids	ND	-	+
Anthocyanidins	ND	-	ND
Alkaloids (Dragendorff)	++	+++	-
Alkaloids (Wagner)	++	-	+++
Alkaloids (Mayer)	-	-	+++
Reducing sugars	ND	+	-
Tannins and phenolic compounds	ND	+c	+f
Mucilages	ND	ND	-

- = Absent; + = Present; ++ = Moderate amount; +++= Highly Present.

$+^f$ = Present in form of phenolic compounds; $+^c$ = Present in form of pyrocatechol tannins.

ND = Not determined.

https://doi.org/10.1371/journal.pone.0319524.t002

ability to neutralize free radicals, prevent lipid peroxidation, and protect against oxidative damage at the cellular level [59,60]. For example, flavonoids detected in the aqueous extract are known to play a vital role in scavenging reactive oxygen species (ROS), thereby mitigating oxidative stress and its associated health implications, such as chronic inflammation and degenerative diseases [61].

The high presence of phenolic compounds in both the alcoholic and aqueous extracts further supports their strong antioxidant potential. The tannins detected in these extracts are also crucial contributors to antioxidant activity, with evidence suggesting their role in chelating metal ions [62,63] and scavenging free radicals effectively neutralizing them and preventing oxidative damage [63,64].

Catechins were detected in the alcoholic extract. Catechins are significant contributors to antioxidant activity due to their ability to scavenge free radicals and inhibit lipid peroxidation [65,66]. Their potency often exceeds that of other polyphenols, making them valuable for health promotion and disease prevention [66,67].

Ethereal extract showed lower levels of antioxidant-related phytochemicals. It primarily contained compounds such as oils and fats, triterpenoids, and steroids. Although these compounds are not direct antioxidants, they contribute to enhancing antioxidant pathways. The ability of triterpenoids and steroids to interact with biological pathways related to lipid metabolism positions them as potential treatments for metabolic disorders [68,69]. They are associated with anti-inflammatory and membrane-protective effects, attributed to their ability to stabilize cell membranes and mitigate oxidative stress [68,70,71].

The absence or non-detection (ND) of certain compounds in specific extracts reflects the limitations of each solvent in extracting compounds beyond its polarity spectrum. This underscores the importance of using multiple extraction methods to comprehensively profile the phytochemical composition of *P. guineense* and to maximize the recovery of compounds with

desired antioxidant activities. The alcoholic extract appears to offer the most balanced profile, combining lipophilic and hydrophilic antioxidants, while the aqueous extract emphasizes hydrophilic compounds with potent ROS-scavenging abilities.

GC-MS analysis

A total of 65 compounds were identified in the extract of *P. guineense* leaves by GC-MS analysis applying different solvents which are shown in Table 3. Among these compounds, alkanes, alkenes, monoterpenes, diterpenes, carboxylic acids, sesquiterpenes, and oxygenated sesquiterpenes were with their peak area values and retention times reported for each solvent. It was experimentally identified that the only sesquiterpene obtained with at least three solvents (hexane, dichloromethane, and ethyl acetate) was alpha-copaene.

Based on the GC-MS data, the solvents yielding the highest percentages of sesquiterpenes were as follows: Hexane (21.6%), Dichloromethane (17.9%), Ethyl Acetate (1.36%), and Methanol (0.60%). Among these, alpha-copaene was the most prominent chemical constituent, accounting for 10.26%, followed by caryophyllene at 6.79%. Other studies performed in Sri Lanka have reported a lower amount of sesquiterpenes (6.1%) in *P. guineense* using hexane as the solvent, with caryophyllene (1.4%) and copaene (1.4%) being the most prominent constituents [72]. In contrast, the only oxygenated sesquiterpene identified with at least two solvents (dichloromethane at 1.66% and ethyl acetate at 5.40%) was gamma-sitosterol. Another study conducted in Egypt, which compared the chemical profiles of different *Psidium guajava* varieties, reported low amounts of caryophyllene oxide (< 0.96%) [73], while for *P. guineense* native of Ecuador, a value of 2.92% was identified in its composition.

The many properties identified in the numerous bioactive compounds are valuable for a range of applications. Copaene has demonstrated antioxidant [74], anticancer, and antigenotoxic effects. Caryophyllene has been reported to possess anticancer, analgesic [74], anti-inflammatory [74], antioxidant [74], antimicrobial [74] activities. There is variability in the quantity and quality of chemical constituents among different *P. guineense* species, which can be attributed to exogenous factors such as climatic conditions [40], and soil properties [72]; in addition to endogenous variables such as physiological [73], genetic [24], and anatomical factors [73].

DNA barcode analysis

PCR amplification was detected for all the nine DNA barcodes tested. The best hit for all sequences for each barcode is indicated (S1 Table). BLAST analysis indicated the presence of *Psidium spp.* using the sequences available in the nr database, including plastid genomes and single locus sequences.

The best model for nucleotide substitution, obtained after alignment of the sequences, which were: T92 (psbA-trnH, psbK-psbI, atpF-atpH, and matK), JC (rpoB, rpoC1, and rbcL), T92+G (ITS1), and K2+G (ITS2). Phylogenetic analysis for psbA-trnH showed that all the *Psidium spp.* are in a clade with a bootstrap value of 90 (Fig 1). For the DNA barcode psbK-psbI, the three biological replicates of *P. guineense* are in a clade with a bootstrap value of 99 (S1 Fig). Furthermore, the atpF-atpH phylogenetic tree showed that the three *P. guineense* from Ecuador are grouped in a clade with other *Psidium* species with a bootstrap value of 93 (S1 Fig). For the *rpoB*, the phylogenetic tree revealed that differentiation between different *Psidium* species and even other genera could not be achieved (Fig 2).

The phylogenetic tree of *rpoC1* showed that the three *P. guineense* are grouped in a clade but due to the absence of *Psidium spp.* sequences in the GenBank for *rpoC1* is not possible to determine genetic diversity between species of the same genera (S1 Fig). The phylogenetic tree with the *rbcL* barcode shows that all accessions from the genus *Psidium* are in one clade,

Table 3. Compounds detected by GC-MS.

Solvents		Hexane		Dichloromethane		Ethyl Acetate		Methanol	
N°	Compound Detected	Peak area (%)	Retention Time (estimated)	Peak area (%)	Retention Time (estimated)	Peak area (%)	Retention Time (estimated)	Peak area (%)	Retention Time (estimated)
1	α-Copaene	10.26	15.174	8.91	11.987	1.36	15.123	Nd	Nd
2	Caryophyllene	6.79	16.319	1.05	15.161	Nd	Nd	0.60	16.294
3	α-Curcumene	1.37	17.814	Nd	Nd	Nd	Nd	Nd	Nd
4	β-Bisabolene	3.18	18.457	Nd	Nd	Nd	Nd	Nd	Nd
5	2, 6, 10-Dodecatrienoic Acid	Nd	24.577	Nd	Nd	Nd	Nd	Nd	Nd
6	Neophytadiene	2.36	25.799	5.04	25.824	2.73	25.760	Nd	Nd
7	Dibutyl Phthalate	2.50	28.063	Nd	Nd	Nd	Nd	Nd	Nd
8	Hexadecanoic Acid	3.14	28.382	2.95	28.445	13.76	28.324	Nd	Nd
9	Phytol	4.54	31.054	Nd	Nd	Nd	Nd	Nd	Nd
10	Nonadecane	1.21	34.413	Nd	Nd	Nd	Nd	Nd	Nd
11	9-Octadecenamide	14.16	35.348	Nd	Nd	Nd	Nd	Nd	Nd
12	Docosane	1.73	36.060	0.61	32.688	Nd	Nd	Nd	Nd
13	Heneicosane	1.77	37.651	Nd	Nd	Nd	Nd	Nd	Nd
14	Hexacosane	2.02	39.178	Nd	Nd	Nd	Nd	Nd	Nd
15	Heptacosane	1.73	40.686	0.76	40.654	Nd	Nd	Nd	Nd
16	Eicosane	1.12	42.079	Nd	Nd	Nd	Nd	Nd	Nd
17	Benzeneacetic Acid	Nd	Nd	2.21	11.987	Nd	Nd	Nd	Nd
18	Alloaromadendrene	Nd	Nd	1.24	17.254	Nd	Nd	Nd	Nd
19	α-Amorphene	Nd	Nd	0.82	17.611	Nd	Nd	Nd	Nd
20	β-Selinene	Nd	Nd	1.06	18.018	Nd	Nd	Nd	Nd
21	α-Muurolene	Nd	Nd	0.60	18.190	Nd	Nd	Nd	Nd
22	γ-Muurolene	Nd	Nd	0.35	18.539	Nd	Nd	Nd	Nd
23	1s,Cis-Calamenene	Nd	Nd	1.22	18.737	Nd	Nd	Nd	Nd
24	α-Calacorene	Nd	Nd	0.87	19.201	Nd	Nd	Nd	Nd
25	Caryophyllene Oxide	Nd	Nd	2.92	20.206	Nd	Nd	Nd	Nd
26	β-Copaen-4-α-Ol	Nd	Nd	1.78	21.650	Nd	Nd	Nd	Nd
27	Copaene	Nd	Nd	1.78	21.650	Nd	Nd	Nd	Nd
28	2-(1,1-dimethylethyl)-4-(1-methyl-1-phenylethyl)phenol,	Nd	Nd	0.63	29.355			Nd	Nd
29	Phytol	Nd	Nd	0.48	31.022	1.64	31.022	Nd	Nd
30	Octadecanoic Acid	Nd	Nd	0.75	32.084	1.85	31.995	Nd	Nd
31	Tricosane	Nd	Nd	0.84	34.406	Nd	Nd	Nd	Nd
32	Eicosanoic Acid	Nd	Nd	0.45	35.437	Nd	Nd	Nd	Nd
33	Tetracosane	Nd	Nd	1.00	36.060	Nd	Nd	Nd	Nd
34	Pentacosane	Nd	Nd	1.06	37.651	Nd	Nd	Nd	Nd
35	Tricosane	Nd	Nd	1.50	39.178	Nd	Nd	Nd	Nd
36	Octacosane	Nd	Nd	1.03	42.072	Nd	Nd	Nd	Nd
37	Triacontane	Nd	Nd	0.28	44.783	Nd	Nd	Nd	Nd
38	Vitamin E	Nd	Nd	0.37	47.232	Nd	Nd	Nd	Nd
39	γ-Sitosterol	Nd	Nd	1.66	48.498	5.40	48.498	Nd	Nd
40	8-Methyl(6)(2,4)Thiophenophane	Nd	Nd	Nd	Nd	2.69	19.615	Nd	Nd
41	(-)-Loliolide	Nd	Nd	Nd	Nd	1.22	24.297	Nd	Nd
42	9-Octadecenoic Acid	Nd	Nd	Nd	Nd	3.15	31.537	Nd	Nd
43	9-Octadecenamide	Nd	Nd	Nd	Nd	7.55	35.310	20.51	35.481
44	Heptanoic Acid	Nd	Nd	Nd	Nd	Nd	Nd	0.05	9.811

(Continued)

Table 3. (Continued)

Solvents		Hexane		Dichloromethane		Ethyl Acetate		Methanol	
N°	Compound Detected	Peak area (%)	Retention Time (estimated)	Peak area (%)	Retention Time (estimated)	Peak area (%)	Retention Time (estimated)	Peak area (%)	Retention Time (estimated)
45	Benzoic Acid Trimethylsilyl Ester	Nd	Nd	Nd	Nd	Nd	Nd	0.34	11.764
46	Butanedioic Acid	Nd	Nd	Nd	Nd	Nd	Nd	0.17	13.558
47	Propanoic Acid	Nd	Nd	Nd	Nd	Nd	Nd	0.10	13.984
48	Butanedioic Acid	Nd	Nd	Nd	Nd	Nd	Nd	0.30	17.992
49	L-Threonic Acid	Nd	Nd	Nd	Nd	Nd	Nd	0.15	19.742
50	Hexadecane	Nd	Nd	Nd	Nd	Nd	Nd	0.08	20.652
51	Dodecanoic Acid	Nd	Nd	Nd	Nd	Nd	Nd	0.14	21.816
52	Benzoic Acid	Nd	Nd	Nd	Nd	Nd	Nd	1.30	25.391
53	Tetradecanoic Acid	Nd	Nd	Nd	Nd	Nd	Nd	0.98	26.040
54	7,9-ditertbutyl-1-oxaspiro [4.5]deca-6,9-diene-2,8-dione	Nd	Nd	Nd	Nd	Nd	Nd	2.26	27.115
55	N-Pentadecanoic Acid	Nd	Nd	Nd	Nd	Nd	Nd	0.11	28.006
56	Oleanitrile	Nd	Nd	Nd	Nd	Nd	Nd	0.83	30.494
57	Octadecoxy-Trimethylsilane	Nd	Nd	Nd	Nd	Nd	Nd	0.66	31.951
58	Hexadecanamide	Nd	Nd	Nd	Nd	Nd	Nd	1.26	32.275
59	Oleic Acid	Nd	Nd	Nd	Nd	Nd	Nd	1.94	32.968
60	11-Trans-Octadecenoic Acid	Nd	Nd	Nd	Nd	Nd	Nd	0.53	33.089
61	Octadecanoic Acid	Nd	Nd	Nd	Nd	Nd	Nd	16.05	33.496
62	Nonadecanoic Acid	Nd	Nd	Nd	Nd	Nd	Nd	0.11	35.125
63	Eicosanoic Acid	Nd	Nd	Nd	Nd	Nd	Nd	0.25	36.735
64	Docosanoic Acid	Nd	Nd	Nd	Nd	Nd	Nd	0.93	39.782
65	Tetracosanoic Acid	Nd	Nd	Nd	Nd	Nd	Nd	0.17	42.613
Total		57.94		44.98		41.35		55.53	
Sesquiterpene		21.6		17.9		1.36		0.60	
Sesquiterpene oxygenated				3.44		5.40			

ND = Not determinated

https://doi.org/10.1371/journal.pone.0319524.t003

indicating that *rbcL* could be used to discriminate between different genera but not different species from the genus *Psidium* (Fig 3). On the other hand, the barcode *matK* could be used to differentiate between different species of *Psidium*, as different clades are formed, especially of *P. guajava* and *P. guineense*, except for the accession of *P. guineense* JQ588513 (Fig 4). The ITS1 barcode showed different clades for different species of *Psidium*, where the *P. guineense* from Ecuador are in clades with 99 and 98 bootstrap values (Fig 5). For the ITS2 barcode, the *P. guineense* from Ecuador are grouped with bootstrap values of 99 and 98 (S1 Fig); however, not all *Psidium* species from the Genbank were clustered in a clade (S1 Fig).

Genetic analysis has emerged as a crucial tool in the standardization of medicinal plants. Characterization of plant species at the genotypic level is essential, as many plants can exhibit significant variations in their physical characteristics even within the same genus and species. DNA analysis is a valuable method for identifying cells, individuals, or species, with the potential to distinguish authentic from adulterated drugs, as discussed by Kumari and Kotecha's study [75]. Various techniques could be employed for genotyping in plants. These include microsatellite markers [76–78], as well as molecular techniques such as genome-wide approaches [79], and transcriptome analysis [80].

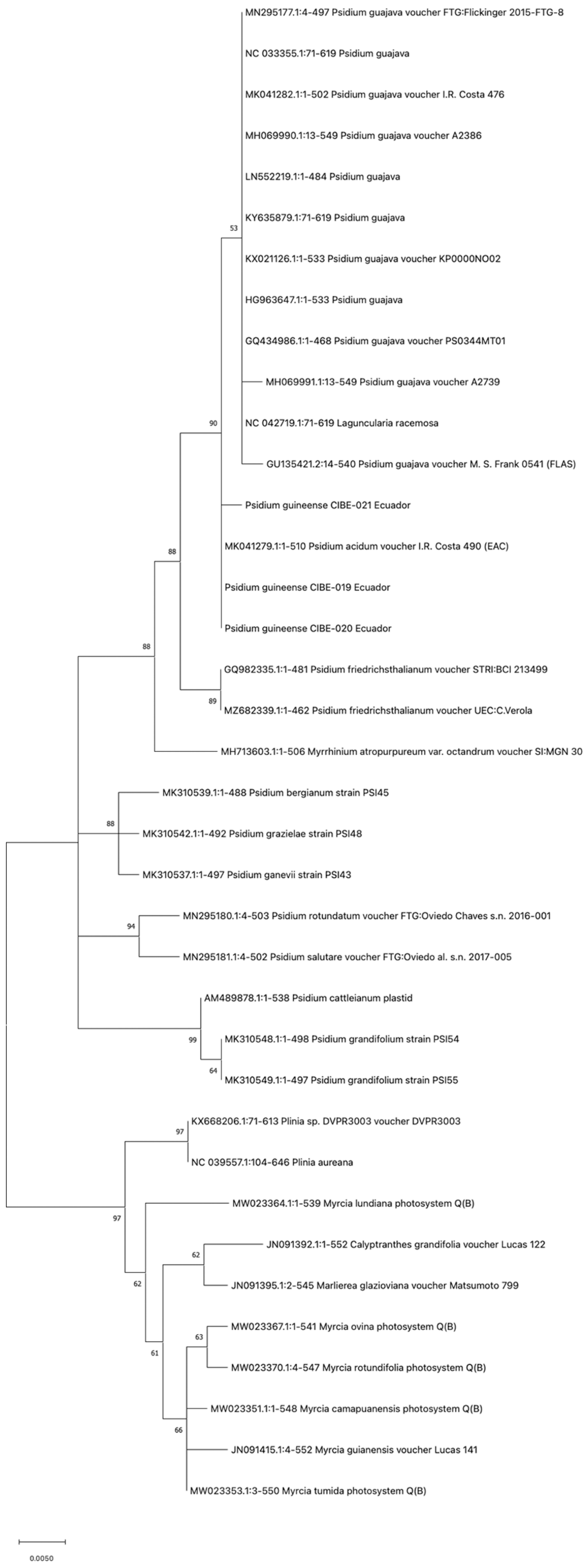

Fig 1. Phylogenetic tree of the *psbA-trnH* barcode with accessions from the genus *Psidium spp*. and different genera selected from the blastn results.

https://doi.org/10.1371/journal.pone.0319524.g001

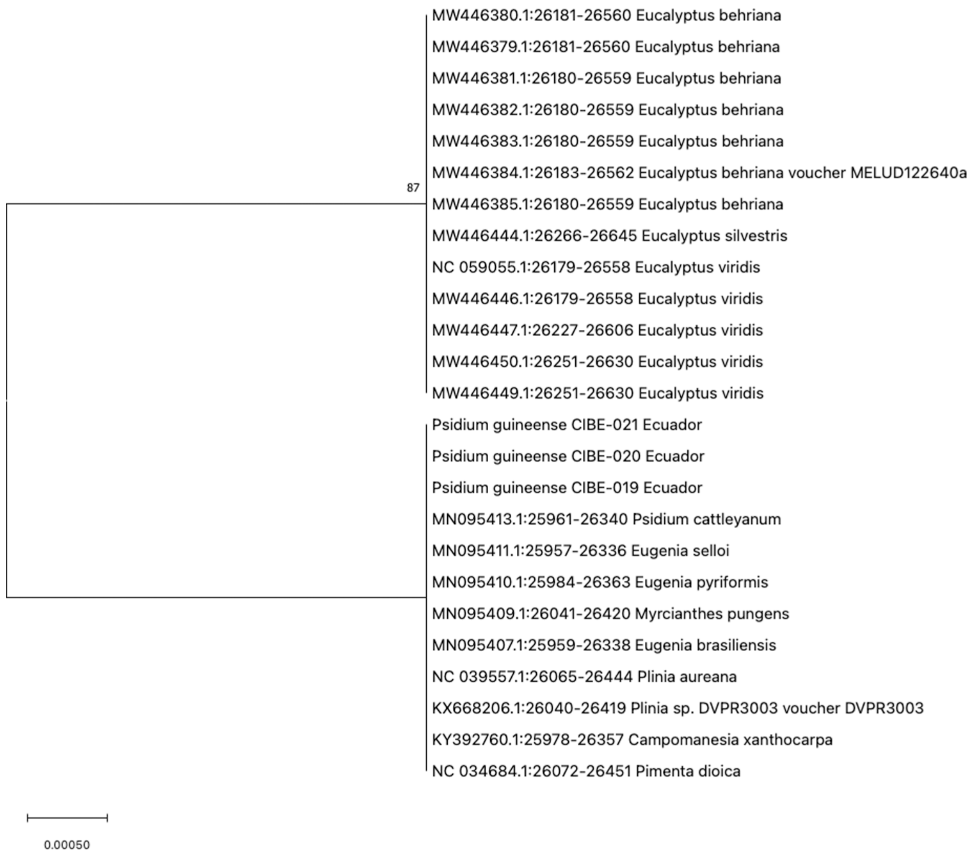

Fig 2. Phylogenetic tree of the *rpoB* barcode with accessions from the genus *Psidium spp*. and different genera selected from the blastn results.

https://doi.org/10.1371/journal.pone.0319524.g002

Our findings indicate that the *rpoB* and *rbcL* barcodes lack accuracy to differentiate at the species level, while for *rpoC1* there are no sequences at the GenBank from *Psidium* species and therefore, could not be concluded that this barcode could be used for different at the species level. For the barcodes *psbA*-trnH, *psbK*-psbI and atpF-*atpH* showed differentiation with other *Psidium* species, although more *Psidium* sequences should be available at the Genbank for a better analysis. The *matK* barcode showed differences at the *Psidium* species but for some species, they did not form a clade.

In contrast, the ITS1 and ITS2 exhibit improved species-level resolution for *Psidium* species. Other reports suggest that ITS2 provides superior resolution for identifying species in medicinal plants [81–85].

Future research efforts should involve the sequencing of specific genetic markers for various *Psidium* species located in Ecuador, while including biological replicates. Subsequent investigations should focus on establishing a robust DNA barcode analysis and exploring various combinations of two genetic *loci* to determine the most effective barcode for species identification within the *Psidium* species.

Total phenolics and flavonoids content and antioxidant activity

Phenolic compounds, such as phenolic acids, tannins, flavonoids, and others, are considered the most important phytochemical compounds produced by plants. These compounds exist

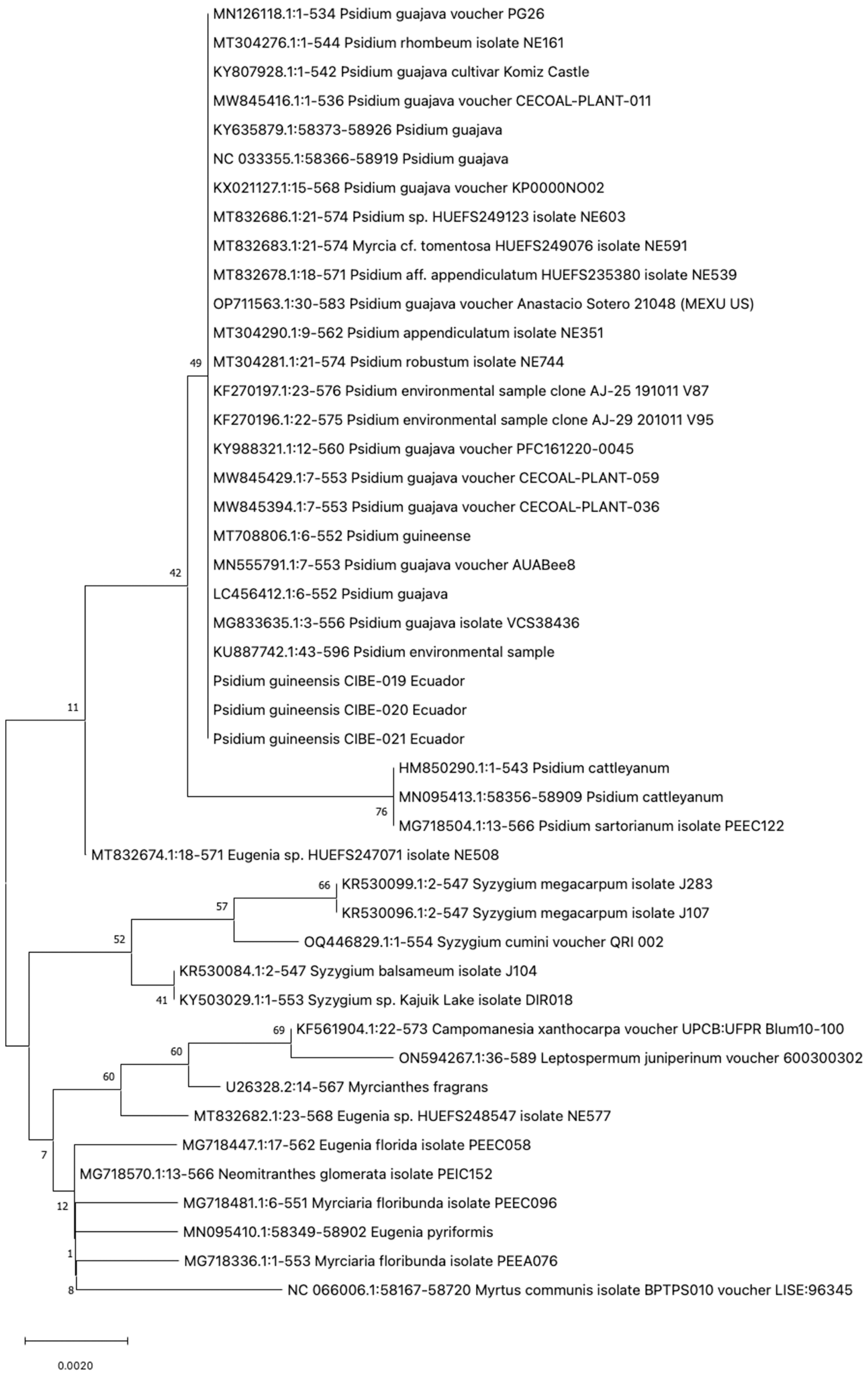

Fig 3. Phylogenetic tree of the *rbcL* barcode with accessions from the genus *Psidium spp*. and different genera selected from the blastn results.

https://doi.org/10.1371/journal.pone.0319524.g003

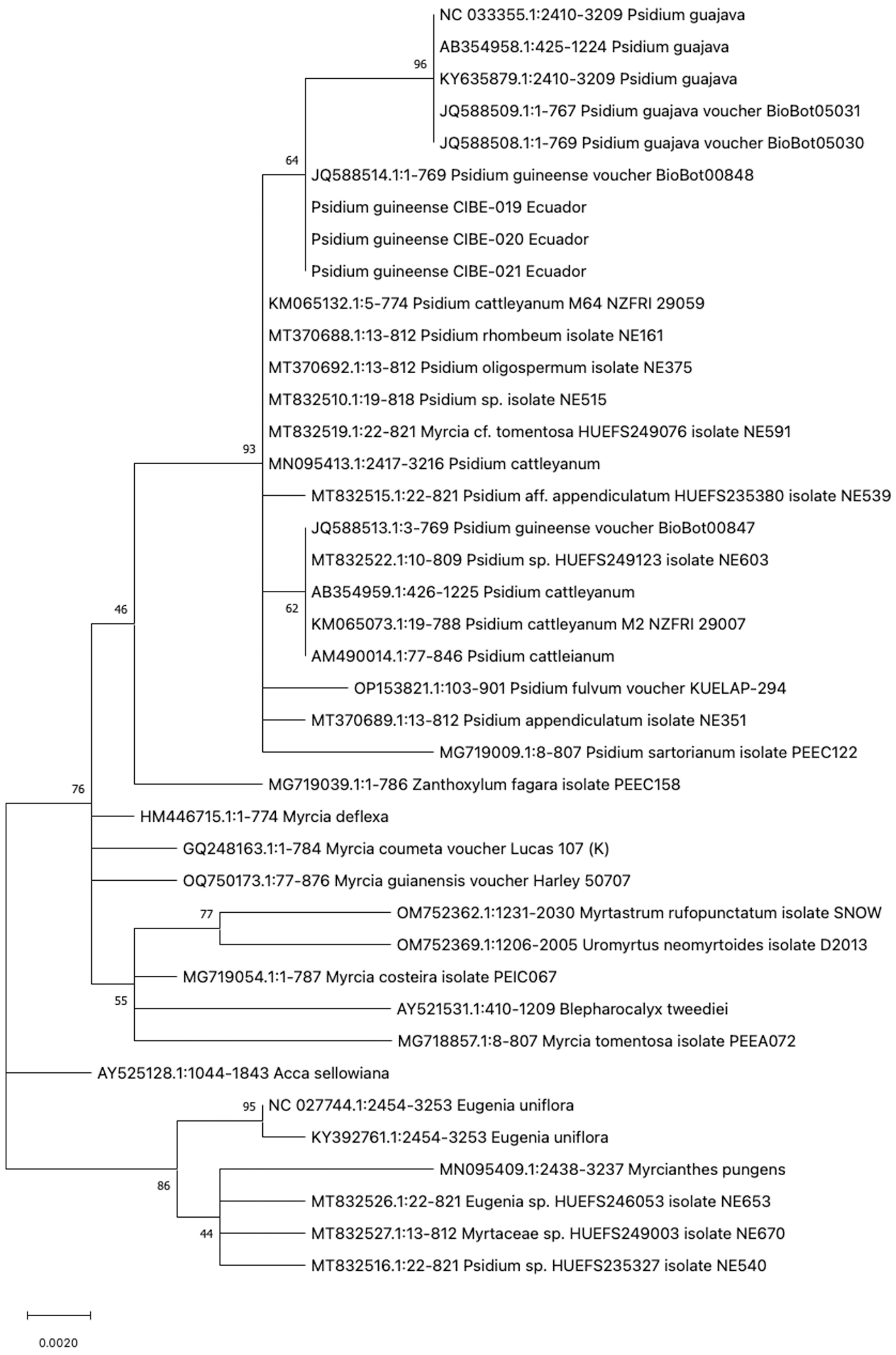

Fig 4. Phylogenetic tree of the *matK* barcode with accessions from the genus *Psidium spp*. and different genera selected from the blastn results.

https://doi.org/10.1371/journal.pone.0319524.g004

in various parts of the plant, and their amounts depend significantly on factors such as the type of plant organ, climate, variety, and location. The leaf extract of *P. guineense* native to the tropical dry forest of Ecuador presents high phenolic and flavonoid content: 54.34 ± 0.49 mg

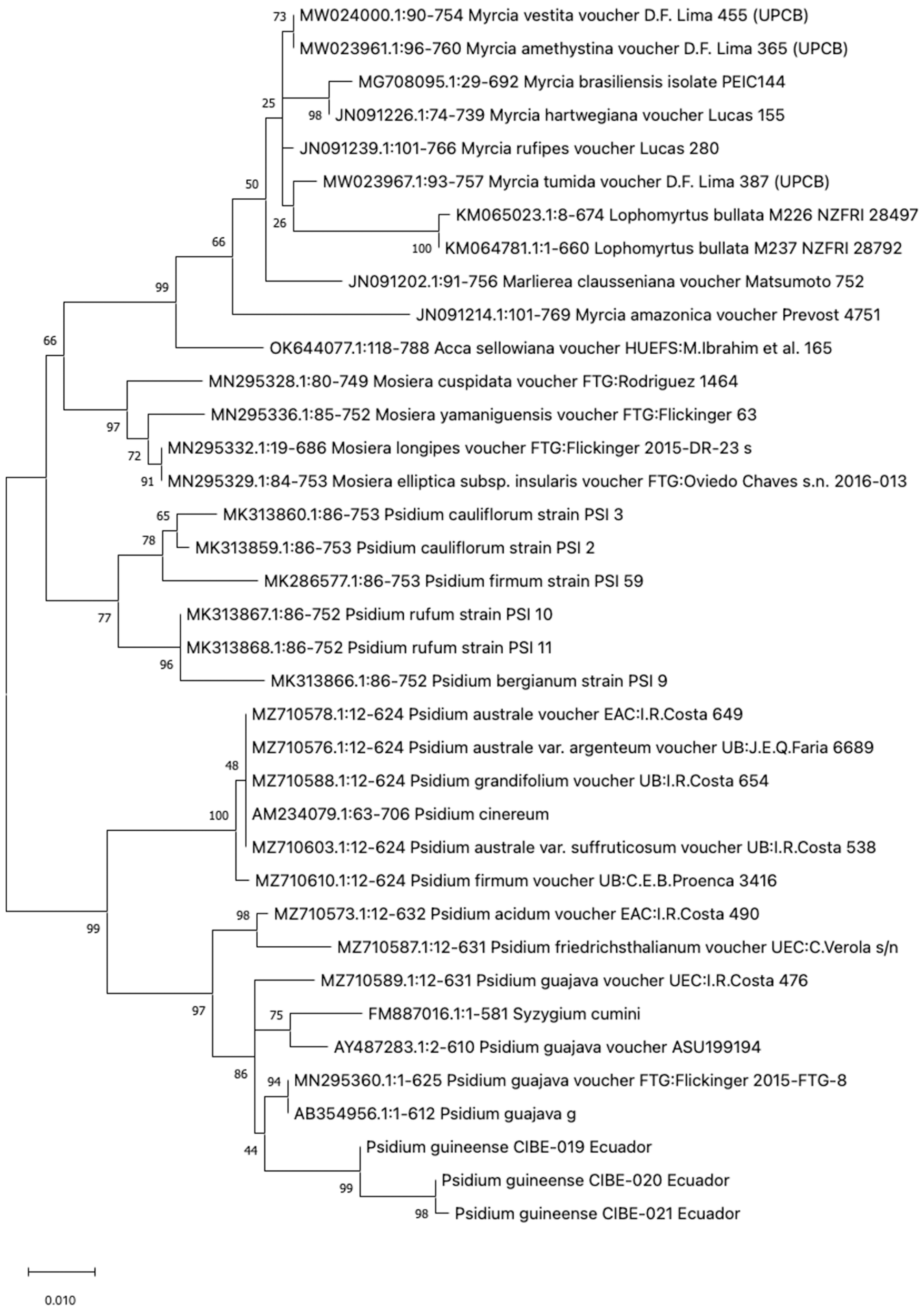

Fig 5. Phylogenetic tree of the ITS1 barcode with accessions from the genus *Psidium spp*. and different genera selected from the blastn results.

https://doi.org/10.1371/journal.pone.0319524.g005

of GAE/g of dried weight and 6.43 ± 0.38 mg QE/g dry weight respectively (Table 4). In a previous study conducted in Ecuador on thirteen native plants of Guayas, phenolic content values of *Psidium guayaquilense* and *Psidium rostratum* leaf extracts were reported, obtaining values of 941.97 ± 30.60 mg GAE/ g dry extract and 591.34 ± 24.31 mg GAE/g dry extract, respectively. However, the *P. guineense* variety was not analyzed [86].

Table 4. Antioxidant activity of *Psidium* species aqueous extracts.

ABTS radical cation inhibition activity (mg TE/g)	DPPH radical scavenging activity (mg TE/g)	Ferric reducing antioxidant power (mg TE/g)	Total phenolic content (mg GAE/g)	Total flavonoid content (mg QE/g)
1.25 ± 0.01	0.57 ± 0.04	105.52 ± 4.84	54.34 ± 0.49	6.43 ± 0.27

https://doi.org/10.1371/journal.pone.0319524.t004

The study carried out in the western province of Sri Lanka reported that the total phenol content in *P. guineense* leaf extract is 195.25 ± 9.56 mg GAE/g [87].

All values are mean ± standard deviation (n=3). ABTS radical cation inhibition activity, DPPH radical scavenging activity, and Ferric reducing antioxidant power values are expressed as mg of Trolox equivalent/g of dried weight. Total phenolic content values are expressed as mg of gallic acid equivalent/g of dried weight. Total flavonoid content values are expressed as mg of quercetin equivalent/g of dried weight. All Data for the calibration curves and results obtained from the samples are available as Supporting Information (S2 Table).

On the other hand, the antioxidant activity of *P. guineense* found in its study is summarized in Table 4, which shows a high antioxidant activity based on the Trolox standard [88]

Conclusion

This study provides valuable insights into the bioactive potential of *Psidium guineense* leaf extract through a comprehensive analysis of its chemical profile, phenolic and flavonoid content, and antioxidant activity. The high phenolic content observed in the extract support the established correlation between phenolic compounds and antioxidant activity, confirming the extract's potential health-promoting properties.

In terms of genetic characterization, the *matK* and ITS1 barcodes have demonstrated their utility in distinguishing *P. guineense* at species levels when compared with available GenBank sequences. However, further studies incorporating additional *Psidium* species area required to evaluate the effectiveness of the *psbK*-psbI, *atpF-atpH*, *rpoC1,* and ITS2 barcodes for species level identification. The *rbcL* barcode, on the other hand, proves to be reliable for differentiation at the genus level.

Supporting information

S1 Table. Blastn analysis for nine different DNA barcodes of *Psidium guineense* plants (CIBE-019, CIBE-020, CIBE-021). Results were ranked for the first two with the highest percentage of identity.
(XLSX)

S1 Fig. Phylogenetic trees of *Psidium guineense* from Ecuador.
(TIF)

S2 Table. Total Phenolic and Flavonoid Content-Antioxidant Activity of *Psidium guineense*.
(XLSX)

Author contributions

Conceptualization: Joel Eduardo Vielma-Puente, Efrén Santos-Ordóñez, Xavier Cornejo.

Data curation: Joel Eduardo Vielma-Puente, Efrén Santos-Ordóñez, Ricardo Pacheco-Coello, Liliana Villao-Uzho, Christian Moreno-Alvarado, Natalia Mendoza-Samaniego, Yuraima Fonseca.

Formal analysis: Joel Eduardo Vielma-Puente, Natalia Mendoza-Samaniego, Yuraima Fonseca.

Investigation: Joel Eduardo Vielma-Puente, Efrén Santos-Ordóñez, Xavier Cornejo, Iván Chóez-Guaranda, Ricardo Pacheco-Coello, Liliana Villao-Uzho, Christian Moreno-Alvarado, Natalia Mendoza-Samaniego, Yuraima Fonseca.

Methodology: Joel Eduardo Vielma-Puente, Efrén Santos-Ordóñez, Xavier Cornejo, Iván Chóez-Guaranda, Ricardo Pacheco-Coello, Liliana Villao-Uzho, Christian Moreno-Alvarado, Natalia Mendoza-Samaniego, Yuraima Fonseca.

Writing – original draft: Joel Eduardo Vielma-Puente, Efrén Santos-Ordóñez, Iván Chóez-Guaranda, Christian Moreno-Alvarado, Natalia Mendoza-Samaniego, Yuraima Fonseca.

Writing – review & editing: Joel Eduardo Vielma-Puente, Efrén Santos-Ordóñez, Natalia Mendoza-Samaniego.

References

1. Mittermeier RA, Turner WR, Larsen FW, Brooks TM, Gascon C. Global Biodiversity Conservation: The Critical Role of Hotspots. Biodiversity Hotspots. Springer Berlin Heidelberg; 2011. pp. 3–22. https://doi.org/10.1007/978-3-642-20992-5_1
2. Kleemann J, Koo H, Hensen I, Mendieta-Leiva G, Kahnt B, Kurze C, et al. Priorities of action and research for the protection of biodiversity and ecosystem services in continental Ecuador. Biological Conservation. 2022;265:109404. https://doi.org/10.1016/j.biocon.2021.109404
3. Cuesta F, Peralvo M, Merino-Viteri A, Bustamante M, Baquero F, Freile JF, et al. Priority areas for biodiversity conservation in mainland Ecuador. Neotropical Biodiversity. 2017;3(1):93–106. https://doi.org/10.1080/23766808.2017.1295705
4. Torri MC. Perceptions and uses of plants for reproductive health among traditional midwives in Ecuador: moving towards intercultural pharmacological practices. Midwifery. 2013;29(7):809–17. https://doi.org/10.1016/j.midw.2012.06.018 PMID: 22877763
5. Nybg, Herbarium S. No Title. 2016. Available from: http://sweetgum.nybg.org/science/vh/specimen-details/?irn=3156114
6. López-Bascón MA, Luque de Castro MD. Soxhlet Extraction. Liquid-Phase Extraction. 2020:327–54. https://doi.org/10.1016/b978-0-12-816911-7.00011-6
7. Valera-Montero LL, Enríquez-Nava S, Silos-Espino H, Padilla-Ramírez JS, Perales Segovia C, Flores-Benítez S. Propiedades fisicoquímicas de guayabilla (Psidium guineense), arrayán (Psidium sartorianum) y guayaba (Psidium guajava). Remexca. 2018;9(6):1099–108. https://doi.org/10.29312/remexca.v9i6.1576
8. Barroso AS, Massing LT, Suemitsu C, Mourão RHV, Figueiredo PLB, Maia JGS. Volatile Constituents of Some Myrtaceous Edible and Medicinal Fruits from the Brazilian Amazon. Foods. 2024;13(10):1490. https://doi.org/10.3390/foods13101490 PMID: 38790790
9. French B, Maynard A. Food Plants International Database. 2022 Nov. Available from: https://foodplantsinternational.com/
10. Eladio J, Loría-Coto M. Illustrative guide to brazilian guava (Psidium guineense). 2023. https://doi.org/10.13140/RG.2.2.11648.92168
11. Lu J, Mao D, Li X, Ma Y, Luan Y, Cao Y, et al. Changes of intestinal microflora diversity in diarrhea model of KM mice and effects of Psidium guajava L. as the treatment agent for diarrhea. J Infect Public Health. 2020;13(1):16–26. https://doi.org/10.1016/j.jiph.2019.04.015 PMID: 31133420
12. Gutiérrez RMP, Mitchell S, Solis RV. Psidium guajava: a review of its traditional uses, phytochemistry and pharmacology. J Ethnopharmacol. 2008;117(1):1–27. https://doi.org/10.1016/j.jep.2008.01.025 PMID: 18353572
13. Durán-Castañeda AC, Cardenas-Castro AP, Pérez-Jiménez J, Pérez-Carvajal AM, Sánchez-Burgos JA, Mateos R, et al. Bioaccessibility of phenolic compounds in Psidium guajava L. varieties and P. friedrichsthalianum Nied. after gastrointestinal digestion. Food Chem. 2023;400:134046. https://doi.org/10.1016/j.foodchem.2022.134046 PMID: 36067696
14. Dike IP, Obembe OO, Adebiyi FE. Ethnobotanical survey for potential anti-malarial plants in south-western Nigeria. J Ethnopharmacol. 2012;144(3):618–26. https://doi.org/10.1016/j.jep.2012.10.002 PMID: 23085021

15. Rajendran C, Begam M, Kumar D, Baruah I, Gogoi HK, Srivastava RB, et al. Antiplasmodial activity of certain medicinal plants against chloroquine resistant Plasmodium berghei infected white albino BALB/c mice. J Parasit Dis. 2014;38(2):148–52. https://doi.org/10.1007/s12639-013-0252-2 PMID: 24808642
16. Amadike Ugbogu E, Emmanuel O, Ebubechi Uche M, Dike Dike E, Chukwuebuka Okoro B, Ibe C, et al. The ethnobotanical, phytochemistry and pharmacological activities of Psidium guajava L. Arabian Journal of Chemistry. 2022;15(5):103759. https://doi.org/10.1016/j.arabjc.2022.103759
17. Machado AJT, Santos ATL, Martins GMAB, Cruz RP, Costa M do S, Campina FF, et al. Antiparasitic effect of the Psidium guajava L. (guava) and Psidium brownianum MART. EX DC. (araçá-de-veado) extracts. Food Chem Toxicol. 2018;119:275–80. https://doi.org/10.1016/j.fct.2018.03.018 PMID: 29548852
18. Flores G, Dastmalchi K, Wu S-B, Whalen K, Dabo AJ, Reynertson KA, et al. Phenolic-rich extract from the Costa Rican guava (Psidium friedrichsthalianum) pulp with antioxidant and anti-inflammatory activity. Potential for COPD therapy. Food Chem. 2013;141(2):889–95. https://doi.org/10.1016/j.foodchem.2013.03.025 PMID: 23790863
19. Medina AL, Haas LIR, Chaves FC, Salvador M, Zambiazi RC, da Silva WP, et al. Araçá (Psidium cattleianum Sabine) fruit extracts with antioxidant and antimicrobial activities and antiproliferative effect on human cancer cells. Food Chemistry. 2011;128(4):916–22. https://doi.org/10.1016/j.foodchem.2011.03.119
20. Pereira E dos S, Vinholes JR, Camargo TM, Nora FR, Crizel RL, Chaves F, et al. Characterization of araçá fruits (Psidium cattleianum Sabine): Phenolic composition, antioxidant activity and inhibition of α-amylase and α-glucosidase. Food Bioscience. 2020;37:100665. https://doi.org/10.1016/j.fbio.2020.100665
21. Felipe do Nascimento K, Leite Kassuya CA, Pereira Cabral MR, Carvalho Souza RI, Marangoni JA, Mussury Franco Silva RM, et al. Chemical analysis and antioxidant, anti-inflammatory and toxicological evaluations of the hydromethanolic extract of Psidium guineense Swartz leaves. J Ethnopharmacol. 2021;281:114492. https://doi.org/10.1016/j.jep.2021.114492 PMID: 34380066
22. Ramos AS, Souza ROS, Boleti AP de A, Bruginski ERD, Lima ES, Campos FR, et al. Chemical characterization and antioxidant capacity of the araçá-pera (Psidium acutangulum): An exotic Amazon fruit. Food Res Int. 2015;75:315–27. https://doi.org/10.1016/j.foodres.2015.06.026 PMID: 28454962
23. Takao LK, Imatomi M, Gualtieri SCJ. Antioxidant activity and phenolic content of leaf infusions of Myrtaceae species from Cerrado (Brazilian Savanna). Braz J Biol. 2015;75(4):948–52. https://doi.org/10.1590/1519-6984.03314 PMID: 26675912
24. Ferreira Macedo JG, de Oliveira Santos M, Nonato C de FA, Torres Salazar GJ, Galvão Rodrigues FF, Almeida-Bezerra JW, et al. Chemical composition, antioxidant, antibacterial and modulating activity of the essential oil of psidium L. species (Myrtaceae Juss.). Biocatalysis and Agricultural Biotechnology. 2022;42:102363. https://doi.org/10.1016/j.bcab.2022.102363
25. dos Santos Rocha T, de Jesus Marques E, do Nascimento CM, Souza RRM, da Costa Silva M, de Souza Neta LC, et al. Chemical and biological profile of Psidium bahianum landrum & funch (Myrtaceae). Braz J Bot. 2021;44(3):537–47. https://doi.org/10.1007/s40415-021-00727-7
26. Halim M, Halim A. The effects of inflammation, aging and oxidative stress on the pathogenesis of diabetes mellitus (type 2 diabetes). Diabetes Metab Syndr. 2019;13(2):1165–72. https://doi.org/10.1016/j.dsx.2019.01.040 PMID: 31336460
27. Abudawood M, Tabassum H, Almaarik B, Aljohi A. Interrelationship between oxidative stress, DNA damage and cancer risk in diabetes (Type 2) in Riyadh, KSA. Saudi J Biol Sci. 2020;27(1):177–83. https://doi.org/10.1016/j.sjbs.2019.06.015 PMID: 31889833
28. Andreadi A, Bellia A, Di Daniele N, Meloni M, Lauro R, Della-Morte D, et al. The molecular link between oxidative stress, insulin resistance, and type 2 diabetes: A target for new therapies against cardiovascular diseases. Curr Opin Pharmacol. 2022;62:85–96. https://doi.org/10.1016/j.coph.2021.11.010 PMID: 34959126
29. Briyal S, Ranjan AK, Gulati A. Oxidative stress: A target to treat Alzheimer's disease and stroke. Neurochem Int. 2023;165:105509. https://doi.org/10.1016/j.neuint.2023.105509 PMID: 36907516
30. Park HS, Kim SR, Lee YC. Impact of oxidative stress on lung diseases. Respirology. 2009;14(1):27–38. https://doi.org/10.1111/j.1440-1843.2008.01447.x PMID: 19144046
31. Demirel Ozbek Y, Saral O, Turker PF. Modern and traditional cooking methods affect the antioxidant activity and phenolic compounds content of Trachystemon Orientalis (L.) G. Don. PLoS One. 2024;19(2):e0299037. https://doi.org/10.1371/journal.pone.0299037 PMID: 38394328

32. Aryee A, Agyei D, Akanbi T. Food for oxidative stress relief: Polyphenols. 2018. https://doi.org/10.1016/B978-0-12-814026-0.21779-4
33. Ferreira Macedo JG, Linhares Rangel JM, de Oliveira Santos M, Camilo CJ, Martins da Costa JG, Maria de Almeida Souza M. Therapeutic indications, chemical composition and biological activity of native Brazilian species from Psidium genus (Myrtaceae): A review. J Ethnopharmacol. 2021;278114248. https://doi.org/10.1016/j.jep.2021.114248 PMID: 34058313
34. Macaúbas-Silva C, Félix MDG, Aquino AKS de, Pereira-Júnior PG, Brito EV de O, Oliveira-Filho AA de, et al. Araçain, a tyrosol derivative and other phytochemicals from Psidium guineense Sw. Nat Prod Res. 2021;35(14):2424–8. https://doi.org/10.1080/14786419.2019.1672683 PMID: 31581838
35. Venkatachalam RN, Singh K, Marar T. Phytochemical screening in vitro antioxidant activity of psidium guajava. Free Radicals and Antioxidants. 2012;2(1):31–6. https://doi.org/10.5530/ax.2012.2.7
36. da Fonsêca BMB, Costa WK, Guimarães Silva VB, Assunção Ferreira MR, Soares LAL, de Oliveira AM, et al. Extract from Psidium guineense Sw leaves: An alternative against resistant strains of Staphylococcus aureus. South African Journal of Botany. 2024;174:850–5. https://doi.org/10.1016/j.sajb.2024.09.017
37. Abrao FY, Costa HM da, Fiuza T de S, Romano CA, Ferreira HD, Cunha LC da, et al. Anatomical study of the leaves and evaluation of the chemical composition of the volatile oils from Psidium guineense Swartz leaves and fruits. RSD. 2021;10(6):e49110615929. https://doi.org/10.33448/rsd-v10i6.15929
38. Fernandes TG, de Mesquita ARC, Randau KP, Franchitti AA, Ximenes EA. In vitro synergistic effect of Psidium guineense (Swartz) in combination with antimicrobial agents against methicillin-resistant Staphylococcus aureus strains. ScientificWorldJournal. 2012;2012:158237. https://doi.org/10.1100/2012/158237 PMID: 22619603
39. da Veiga Correia VT, da Silva PR, Ribeiro CMS, Ramos ALCC, Mazzinghy AC do C, Silva VDM, et al. An Integrative Review on the Main Flavonoids Found in Some Species of the Myrtaceae Family: Phytochemical Characterization, Health Benefits and Development of Products. Plants (Basel). 2022;11(20):2796. https://doi.org/10.3390/plants11202796 PMID: 36297820
40. do Nascimento KF, Moreira FMF, Alencar Santos J, Kassuya CAL, Croda JHR, Cardoso CAL, et al. Antioxidant, anti-inflammatory, antiproliferative and antimycobacterial activities of the essential oil of Psidium guineense Sw. and spathulenol. J Ethnopharmacol. 2018;210:351–8. https://doi.org/10.1016/j.jep.2017.08.030 PMID: 28844678
41. Safhi FA, Alshamrani SM, Bogmaza AFM, El-Moneim DA. DNA Barcoding of Wild Plants with Potential Medicinal Properties from Faifa Mountains in Saudi Arabia. Genes (Basel). 2023;14(2):469. https://doi.org/10.3390/genes14020469 PMID: 36833396
42. Cornejo X. Specimen Details - The William & Lynda Steere Herbarium. In: New York Botanical Garden [Internet]. 6 Jun 2015 [cited 5 Jun 2024]. Available from: https://sweetgum.nybg.org/science/vh/specimen-details/?irn=3156114
43. Jackson BD. A Glossary of Botanic Terms with their Derivation and Accent. Nature. 1900;63(1619):28–28. https://doi.org/10.1038/063028b0
44. López-Bascón MA MD, L de C. Soxhlet Extraction. 1st ed. In: Poole CF, editor. Liquid-phase extraction. 1st ed. Amsterdam, Netherlands: Elsevier; 2020. p. 796.
45. Cuéllar Cuéllar A. y Miranda Martínez M. Farmacognosia y productos naturales. 1st ed. La Habana: Empresa Editorial Poligráfica Félix Varela; 2014.
46. Nasrollahi S, Ghoreishi SM, Ebrahimabadi AH, Khoobi A. Gas chromatography-mass spectrometry analysis and antimicrobial, antioxidant and anti-cancer activities of essential oils and extracts of Stachys schtschegleevii plant as biological macromolecules. Int J Biol Macromol. 2019;128:718–23. https://doi.org/10.1016/j.ijbiomac.2019.01.165 PMID: 30708000
47. Pacheco Coello R, Pestana Justo J, Factos Mendoza A, Santos Ordoñez E. Comparison of three DNA extraction methods for the detection and quantification of GMO in Ecuadorian manufactured food. BMC Res Notes. 2017;10(1):758. https://doi.org/10.1186/s13104-017-3083-x PMID: 29262852
48. Chen S, Yao H, Han J, Liu C, Song J, Shi L, et al. Validation of the ITS2 region as a novel DNA barcode for identifying medicinal plant species. PLoS One. 2010;5(1):e8613. https://doi.org/10.1371/journal.pone.0008613 PMID: 20062805
49. Basak S, Aadi Moolam R, Parida A, Mitra S, Rangan L. Evaluation of rapid molecular diagnostics for differentiating medicinal Kaempferia species from its adulterants. Plant Divers. 2019;41(3):206–11. https://doi.org/10.1016/j.pld.2019.04.003 PMID: 31453420

50. Costion C, Ford A, Cross H, Crayn D, Harrington M, Lowe A. Plant DNA barcodes can accurately estimate species richness in poorly known floras. PLoS One. 2011;6(11):e26841. https://doi.org/10.1371/journal.pone.0026841 PMID: 22096501
51. Stecher G, Tamura K, Kumar S. Molecular Evolutionary Genetics Analysis (MEGA) for macOS. Mol Biol Evol. 2020;37(4):1237–9. https://doi.org/10.1093/molbev/msz312 PMID: 31904846
52. Zhang Z, Schwartz S, Wagner L, Miller W. A greedy algorithm for aligning DNA sequences. J Comput Biol. 2000;7(1–2):203–14. https://doi.org/10.1089/10665270050081478 PMID: 10890397
53. Avramova V, AbdElgawad H, Vasileva I, Petrova AS, Holek A, Mariën J, et al. High Antioxidant Activity Facilitates Maintenance of Cell Division in Leaves of Drought Tolerant Maize Hybrids. Front Plant Sci. 2017;884. https://doi.org/10.3389/fpls.2017.00084 PMID: 28210264
54. Viteri R, Giordano A, Montenegro G, Zacconi F. Eucryphia cordifolia extracts: Phytochemical screening, antibacterial and antioxidant activities. Nat Prod Res. 2022;36(16):4177–81. https://doi.org/10.1080/14786419.2021.1960525 PMID: 34448426
55. Hozzein WN, Saleh AM, Habeeb TH, Wadaan MAM, AbdElgawad H. CO2 treatment improves the hypocholesterolemic and antioxidant properties of fenugreek seeds. Food Chem. 2020;308:125661. https://doi.org/10.1016/j.foodchem.2019.125661 PMID: 31669948
56. Santos JRA, Pereira MLA, Pereira TCJ, Silva HGO, Santos OO, Carvalho GGP, et al. Supplementation with mesquite alkaloids extract in diets for lambs fed Bermuda grass improves growth performance. Small Ruminant Research. 2021;205:106560. https://doi.org/10.1016/j.smallrumres.2021.106560
57. de Araújo RL, de Pinho CLC, Farias FO, Igarashi-Mafra L, Mafra MR. Crinum L. species as a potential source of alkaloids: Extraction methods and relevance for medicinal and pharmacological applications. South African Journal of Botany. 2022;151:720–34. https://doi.org/10.1016/j.sajb.2022.10.053
58. Gañán J, Martínez-García G, Morante-Zarcero S, Pérez-Quintanilla D, Sierra I. Nanomaterials-modified electrochemical sensors for sensitive determination of alkaloids: Recent trends in the application to biological, pharmaceutical and agri-food samples. Microchemical Journal. 2023;184:108136. https://doi.org/10.1016/j.microc.2022.108136
59. Formagio ASN, Volobuff CRF, Santiago M, Cardoso CAL, Vieira M do C, Valdevina Pereira Z. Evaluation of Antioxidant Activity, Total Flavonoids, Tannins and Phenolic Compounds in Psychotria Leaf Extracts. Antioxidants (Basel). 2014;3(4):745–57. https://doi.org/10.3390/antiox3040745 PMID: 26785238
60. Kopjar M, Tadić M, Piližota V. Phenol content and antioxidant activity of green, yellow and black tea leaves. Chem Biol Techn Agric. 2015;2(1):1. https://doi.org/10.1186/s40538-014-0028-7
61. Tungmunnithum D, Thongboonyou A, Pholboon A, Yangsabai A. Flavonoids and Other Phenolic Compounds from Medicinal Plants for Pharmaceutical and Medical Aspects: An Overview. Medicines (Basel). 2018;5(3):93. https://doi.org/10.3390/medicines5030093 PMID: 30149600
62. Abdul M, Taleb M, Asadujjaman M, Tabassum F, Harun M. A review study on the pharmacological effects and mechanism of action of tannins. European Journal of Pharmaceutical and Medical Research. 2021;8. Available from: https://www.researchgate.net/publication/354163529
63. Tong Z, He W, Fan X, Guo A. Biological Function of Plant Tannin and Its Application in Animal Health. Front Vet Sci. 2022;8:803657. https://doi.org/10.3389/fvets.2021.803657 PMID: 35083309
64. Sieniawska E. Activities of tannins-from in vitro studies to clinical trials. Natural Products Communications. 2015;10(11).
65. Munteanu IG, Apetrei C. Assessment of the Antioxidant Activity of Catechin in Nutraceuticals: Comparison between a Newly Developed Electrochemical Method and Spectrophotometric Methods. Int J Mol Sci. 2022;23(15):8110. https://doi.org/10.3390/ijms23158110 PMID: 35897695
66. Nain CW, Mignolet E, Herent M-F, Quetin-Leclercq J, Debier C, Page MM, et al. The Catechins Profile of Green Tea Extracts Affects the Antioxidant Activity and Degradation of Catechins in DHA-Rich Oil. Antioxidants (Basel). 2022;11(9):1844. https://doi.org/10.3390/antiox11091844 PMID: 36139917
67. Sutherland BA, Rahman RMA, Appleton I. Mechanisms of action of green tea catechins, with a focus on ischemia-induced neurodegeneration. J Nutr Biochem. 2006;17(5):291–306. https://doi.org/10.1016/j.jnutbio.2005.10.005 PMID: 16443357
68. Saher S, Rehman MH-U-, Imran M, Nadeem M, Abbas F, Atta Khan F, et al. Fatty acids profile, antioxidant activity, lipid oxidation, induction period, and sensory properties of burgers produced from blends of fish and mango kernel oils. International Journal of Food Properties. 2023;26(2):2811–25. https://doi.org/10.1080/10942912.2023.2252206

69. Taghvaei M, Jafari SM. Application and stability of natural antioxidants in edible oils in order to substitute synthetic additives. J Food Sci Technol. 2015;52(3):1272–82. https://doi.org/10.1007/s13197-013-1080-1 PMID: 25745196

70. Jordamovc N, Pehlivanovic B, Niksic H, Gusic I, Koric E, Dedic M, et al. Anti-proliferative and anti-inflammatory activity of triterpene extracts from plant species belonging to Lamiaceae family. BLACPMA. 2023;22(6):864–78. https://doi.org/10.37360/blacpma.23.22.6.58

71. Nguyen T-D, Nguyen T-H-A, Do T-H, Tran VT-H, Nguyen H-A, Pham D-V. Anti-inflammatory effect of a triterpenoid from Balanophora laxiflora: results of bioactivity-guided isolation. Heliyon. 2022;8(3):e09070. https://doi.org/10.1016/j.heliyon.2022.e09070 PMID: 35287327

72. Kokilananthan S, Bulugahapitiya VP, Manawadu H, Gangabadage CS. Sesquiterpenes and monoterpenes from different varieties of guava leaf essential oils and their antioxidant potential. Heliyon. 2022;8(12):e12104. https://doi.org/10.1016/j.heliyon.2022.e12104 PMID: 36568663

73. Hassan EM, El Gendy AE-NG, Abd-ElGawad AM, Elshamy AI, Farag MA, Alamery SF, et al. Comparative Chemical Profiles of the Essential Oils from Different Varieties of Psidium guajava L. Molecules. 2020;26(1):119. https://doi.org/10.3390/molecules26010119 PMID: 33383905

74. Hajam TA, H S. Phytochemistry, biological activities, industrial and traditional uses of fig (Ficus carica): A review. Chem Biol Interact. 2022;368:110237. https://doi.org/10.1016/j.cbi.2022.110237 PMID: 36288779

75. Singh R, Kotecha M. A review on the Standardization of herbal medicines. Article in International Journal of Pharma Sciences and Research. 2016. Available from: https://www.researchgate.net/publication/298426911

76. Rai MK, Phulwaria M, Shekhawat NS. Transferability of simple sequence repeat (SSR) markers developed in guava (*Psidium guajava L.*) to four Myrtaceae species. Mol Biol Rep. 2013;40(8):5067–71. https://doi.org/10.1007/s11033-013-2608-1 PMID: 23657599

77. Urquía D, Gutierrez B, Pozo G, Pozo MJ, Espín A, Torres M de L. Psidium guajava in the Galapagos Islands: Population genetics and history of an invasive species. PLoS One. 2019;14(3):e0203737. https://doi.org/10.1371/journal.pone.0203737 PMID: 30865637

78. Kumar C, Kumar R, Singh SK, Goswami AK, Nagaraja A, Paliwal R, et al. Development of novel g-SSR markers in guava (Psidium guajava L.) cv. Allahabad Safeda and their application in genetic diversity, population structure and cross species transferability studies. PLoS One. 2020;15(8):e0237538. https://doi.org/10.1371/journal.pone.0237538 PMID: 32804981

79. Thakur S, Yadav IS, Jindal M, Sharma PK, Dhillon GS, Boora RS, et al. Development of Genome-Wide Functional Markers Using Draft Genome Assembly of Guava (Psidium guajava L.) cv. Allahabad Safeda to Expedite Molecular Breeding. Front Plant Sci. 2021;12:708332. https://doi.org/10.3389/fpls.2021.708332 PMID: 34630458

80. Mittal A, Yadav IS, Arora NK, Boora RS, Mittal M, Kaur P, et al. RNA-sequencing based gene expression landscape of guava cv. Allahabad Safeda and comparative analysis to colored cultivars. BMC Genomics. 2020;21(1):484. https://doi.org/10.1186/s12864-020-06883-6 PMID: 32669108

81. Techen N, Parveen I, Pan Z, Khan IA. DNA barcoding of medicinal plant material for identification. Curr Opin Biotechnol. 2014;25103–10. https://doi.org/10.1016/j.copbio.2013.09.010 PMID: 24484887

82. Zhang D, Jiang B, Duan L, Zhou N. Internal transcribed spacer (ITS), an ideal DNA barcode for species discrimination in crawfurdia wall. (GENTIANACEAE). Afr J Tradit Complement Altern Med. 2016;101–106. https://doi.org/10.21010/ajtcam

83. Bustamante K, Santos-Ordóñez E, Miranda M, Pacheco R, Gutiérrez Y, Scull R. Morphological and molecular barcode analysis of the medicinal tree Mimusops coriacea (A.DC.) Miq. collected in Ecuador. PeerJ. 2019;7:e7789. https://doi.org/10.7717/peerj.7789 PMID: 31616590

84. Sarmiento-Tomalá G, Santos-Ordóñez E, Miranda-Martínez M, Pacheco-Coello R, Scull-Lizama R, Gutiérrez-Gaitén Y, et al. Short Communication: Molecular barcode and morphology analysis of Malva pseudolavatera Webb & Berthel and Malva sylvestris L. from Ecuador. Biodiversitas. 2020;21(8). https://doi.org/10.13057/biodiv/d210818

85. Soledispa P, Santos-Ordóñez E, Miranda M, Pacheco R, Gutiérrez Gaiten YI, Scull R. Molecular barcode and morphological analysis of Smilax purhampuy Ruiz, Ecuador. PeerJ. 2021;9:e11028. https://doi.org/10.7717/peerj.11028 PMID: 33777526

86. María R, Shirley M, Xavier C, Jaime S, David V, Rosa S, et al. Preliminary phytochemical screening, total phenolic content and antibacterial activity of thirteen native species from Guayas province Ecuador. Journal of King Saud University - Science. 2018;30(4):500–5. https://doi.org/10.1016/j.jksus.2017.03.009

87. Senanayake CM, Hapugaswatta H, Jayathilaka N, Seneviratne KN. Phenolic extracts of the leaves ofPsidium guineenseSw. improve the shelf life of sunflower oil and baked cake and antioxidant status of Wistar rats. J Food Biochem. 2018;42(6). https://doi.org/10.1111/jfbc.12632

88. Wołosiak R, Drużyńska B, Derewiaka D, Piecyk M, Majewska E, Ciecierska M, et al. Verification of the Conditions for Determination of Antioxidant Activity by ABTS and DPPH Assays-A Practical Approach. Molecules. 2021;27(1):50. https://doi.org/10.3390/molecules27010050 PMID: 35011274
